# Supplementary material for: Antibody Binding Captures High Energy State of an Antigen: The Case of Nsp1 SARS-CoV-2 as Revealed by Hydrogen–Deuterium Exchange Mass Spectrometry
Source: Int J Mol Sci. 2023 Dec 11;24(24):17342. doi: 10.3390/ijms242417342 (PMC10743928; doi:10.3390/ijms242417342)
Supplement: Supplementary file 1 [file ijms-24-17342-s001.zip › ijms-2736266-supplementary.pdf]

Supplementary Information

Antibody Binding Captures High Energy State of an Antigen: The Case of Nsp1 SARS-CoV-2 as Revealed by Hydrogen-Deuterium Exchange Mass Spectrometry

Ravi Kant[1,2], Nawneet Mishra[3], Michael L. Gross[2*]

1University School of Biotechnology, Guru Gobind Singh Indra Prastha University, Dwarka, Delhi, 110078, India

2Department of Chemistry, Washington University, St Louis, MO 63130, USA.

3Department of Pathology and Immunology, Washington University School of Medicine in St Louis, St Louis, MO 63110, USA.

* Correspondence: mgross@wustl.edu

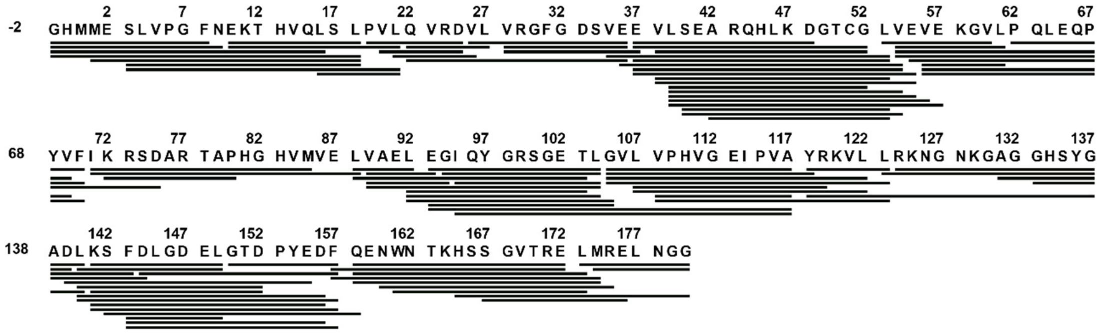

Figure S1. Coverage map of all peptides obtained for the LukF after double digestion, Fungal XIII followed by pepsin. The horizontal bar represents peptides spanning different segments

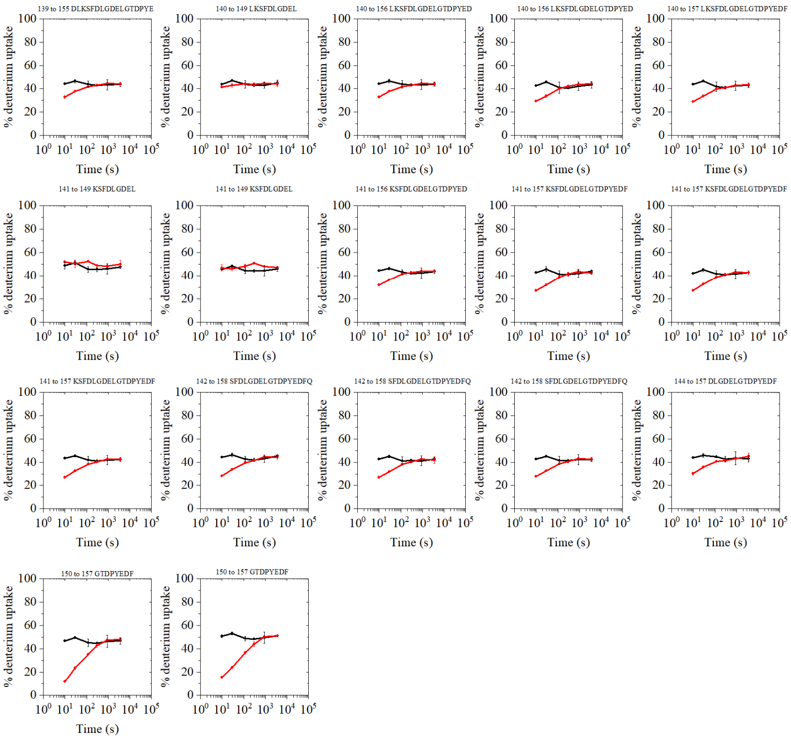

Figure S2. HDX kinetics curves of peptides exhibiting HDX differences from the putative binding site towards the C-terminus of full length Nsp1: unbound (black) and antibody-bound (15497).

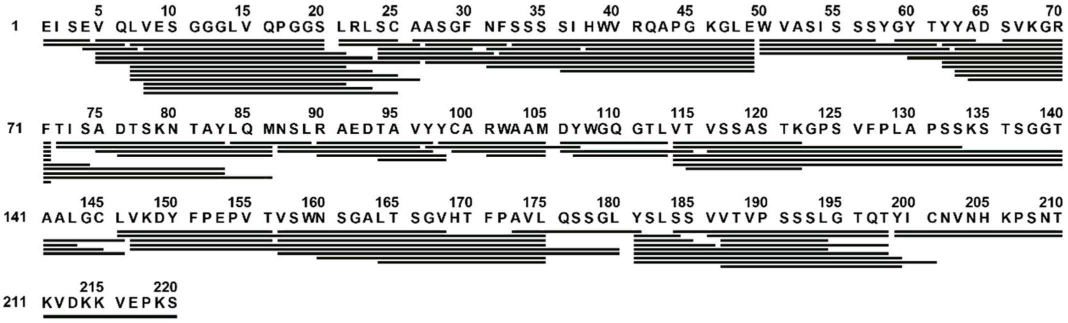

A

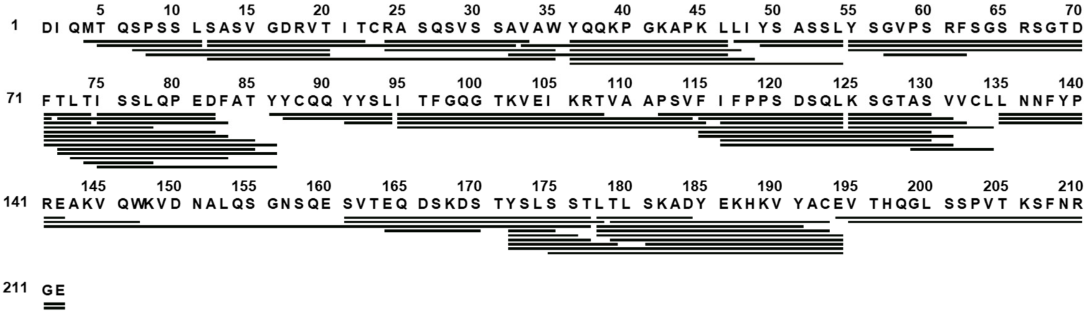

B

Figure S3. Coverage map of all peptides obtained for the antibody 15497 A) Heavy Chain B) Light chain after double digestion, Fungal XIII followed by pepsin. The horizontal bar represents peptides spanning different segments of the sequence.

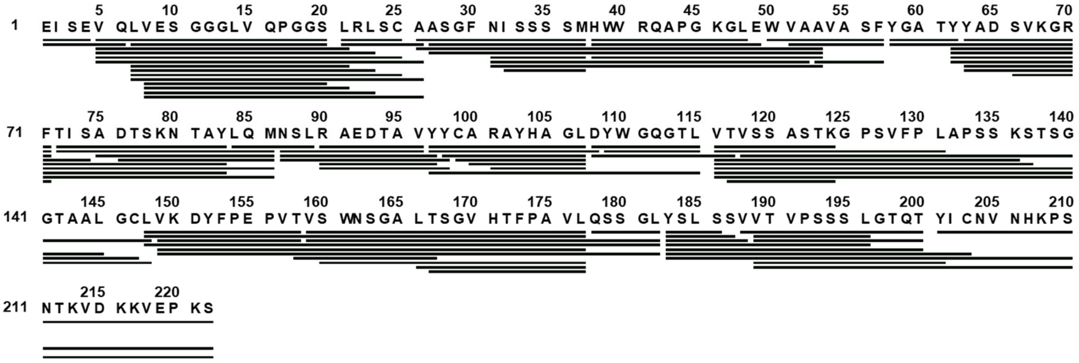

A

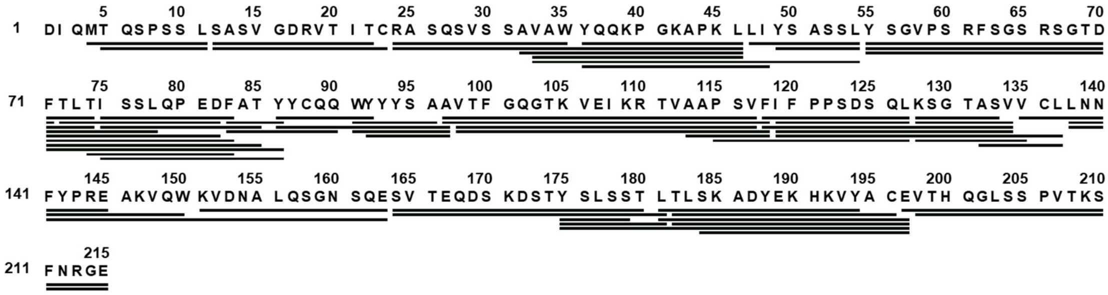

B

Figure S4. Coverage map of all peptides obtained for the antibody 15498 A) Heavy Chain B) Light chain after double digestion, Fungal XIII followed by pepsin. The horizontal bar represents peptides spanning different segments of the sequence.

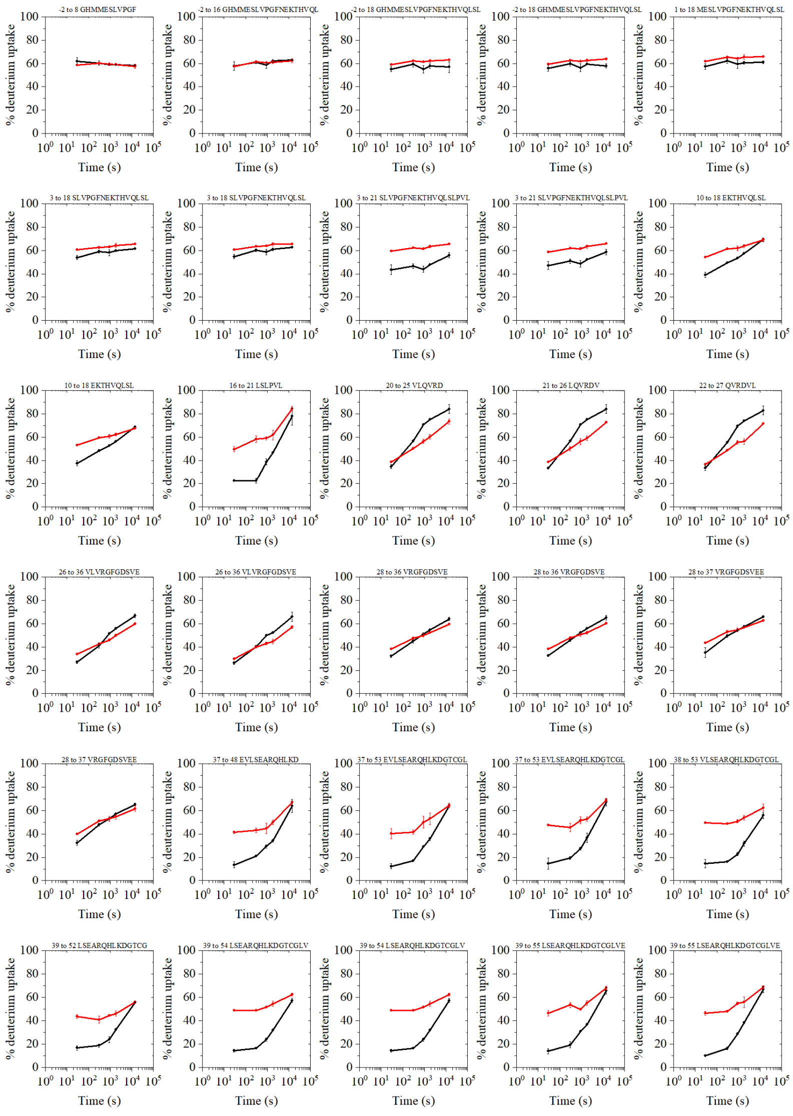

Figure S5. HDX kinetics curves of peptides from N terminal domain of full length Nsp1, unbound (black), bound to antibody (15498).

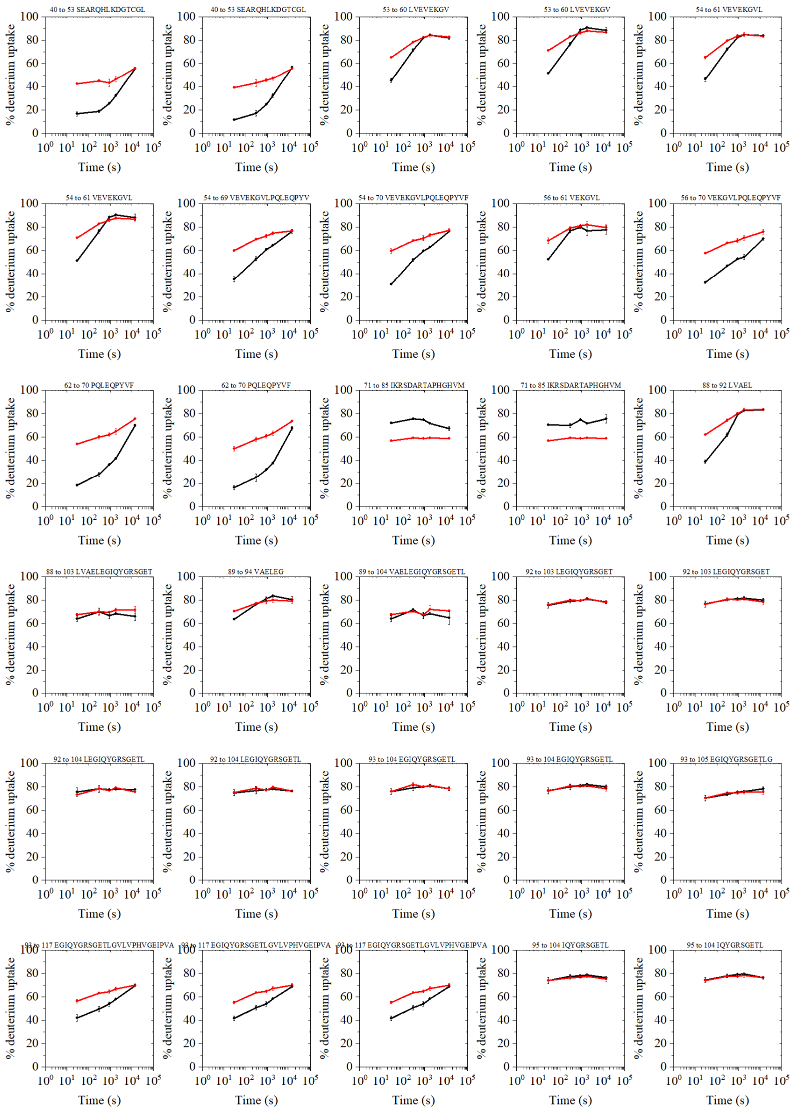

Figure S5. HDX kinetics curves of peptides from N terminal domain of full length Nsp1, unbound (black), bound to antibody (15498).

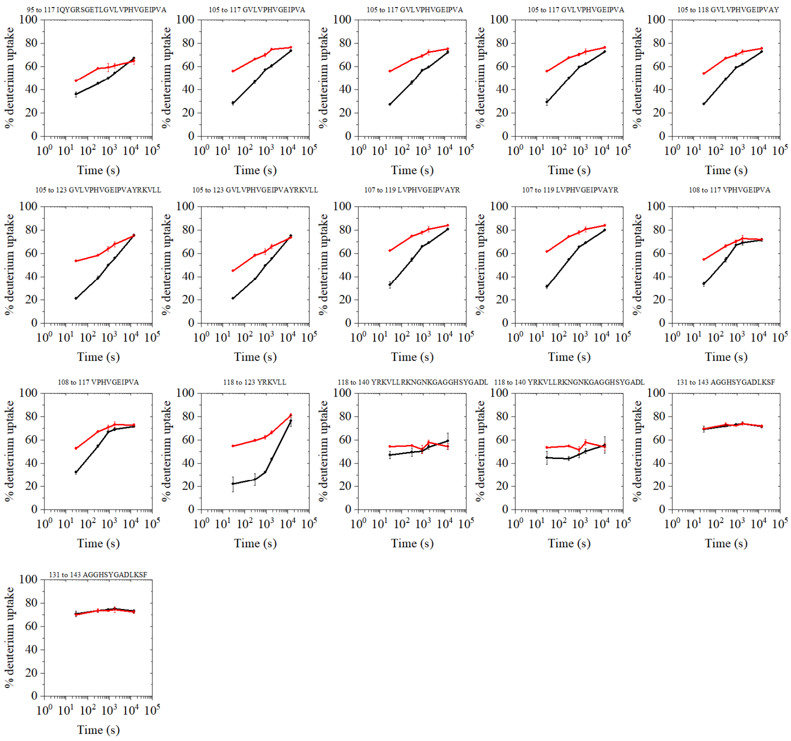

Figure S5. HDX kinetics curves of peptides from N terminal domain of full length Nsp1, unbound (black), bound to antibody (15498).
